# A Different View of Solar Spectral Irradiance Variations: Modeling Total Energy over Six-Month Intervals

**DOI:** 10.1007/s11207-015-0766-0

**Published:** 2015-09-09

**Authors:** Thomas N. Woods, Martin Snow, Jerald Harder, Gary Chapman, Angela Cookson

**Affiliations:** Laboratory for Atmospheric and Space Physics (LASP), University of Colorado, 3665 Discovery Dr., Boulder, CO 80303 USA; San Fernando Observatory (SFO), California State University Northridge, 14031 San Fernando Rd., Sylmar, CA 91342 USA

**Keywords:** Solar spectral irradiance, Solar cycle variations, Irradiance modeling

## Abstract

A different approach to studying solar spectral irradiance (SSI) variations, without the need for long-term (multi-year) instrument degradation corrections, is examining the total energy of the irradiance variation during 6-month periods. This duration is selected because a solar active region typically appears suddenly and then takes 5 to 7 months to decay and disperse back into the quiet-Sun network. The solar outburst energy, which is defined as the irradiance integrated over the 6-month period and thus includes the energy from all phases of active region evolution, could be considered the primary cause for the irradiance variations. Because solar cycle variation is the consequence of multiple active region outbursts, understanding the energy spectral variation may provide a reasonable estimate of the variations for the 11-year solar activity cycle. The moderate-term (6-month) variations from the *Solar Radiation and Climate Experiment* (SORCE) instruments can be decomposed into positive (in-phase with solar cycle) and negative (out-of-phase) contributions by modeling the variations using the San Fernando Observatory (SFO) facular excess and sunspot deficit proxies, respectively. These excess and deficit variations are fit over 6-month intervals every 2 months over the mission, and these fitted variations are then integrated over time for the 6-month energy. The dominant component indicates which wavelengths are in-phase and which are out-of-phase with solar activity. The results from this study indicate out-of-phase variations for the 1400 – 1600 nm range, with all other wavelengths having in-phase variations.

## Introduction

The solar spectral irradiance (SSI) varies on all time scales, and these variations are highly dependent on wavelength. The short-term (days) variations are best understood from observations obtained from several different satellites over the past five decades. There has also been much progress in understanding the longer-term 11-year solar activity cycle variations. However, instrument degradation corrections can be difficult to access and estimating the accuracy of these corrections is important for solar cycle studies. For example, in the near-ultraviolet (NUV: 300 – 400 nm), measurements by the *Solar Radiation and Climate Experiment* (SORCE) have more NUV solar cycle variation than earlier observations (Harder *et al.*^2009^; Unruh, Ball, and Krivova 2012; DeLand and Cebula 2012). These variations have been debated on the basis that they are inconsistent with some SSI model estimates (*e.g.*, Ball *et al.*^2011^; Pagaran *et al.*2011a). Furthermore, some out-of-phase solar cycle variation for various visible (Vis: 400 – 750 nm) and near infrared (NIR: 750 – 1600 nm) wavelengths reported by Harder *et al.* (2009) are not reproduced in these particular model studies. The primary concerns are understanding the solar cycle variation measurement accuracy (total uncertainty) and how much of the NUV–Vis–NIR variations during the decline of Solar Cycle 23 are the consequence of uncorrected instrument degradation trends. While we continue to address the uncertainties of the current and past SSI observations, the focus for this paper is modeling moderate-term (months) solar variations to help improve understanding of solar cycle variations.

There are two primary methods of modeling the SSI variability. One method is purely empirical that relates indices of solar variability to the measured SSI variability, and this method is sometimes referred to as proxy modeling. The Naval Research Laboratory SSI (NRLSSI) model by Lean *et al*. (1997, 2005) is an example of modeling the SSI variability using the sunspot area and Mg ii core-to-wing index (Mg C/W) as proxies for darkening effects of sunspots and brightening effects of faculae/plages. These dark and bright components of variability are critical for the NUV–Vis–NIR and middle ultraviolet (MUV: 200 – 300 nm) ranges, but only the bright component is needed for the soft X-ray (SXR: 0.1 – 10 nm), extreme ultraviolet (EUV: 10 – 120 nm), and far ultraviolet (FUV: 120 – 200 nm). To help improve the accuracy of proxy models for the UV ranges, the 27-day solar rotation variability and 11-year solar cycle variability can be modeled as separate components, such as done for NRLSSI 3C (Lean *et al.*^2011^) and Flare Irradiance Spectral Model (FISM) (Chamberlin, Woods, and Eparvier, 2007, 2008). The need for these two variability components is related to how important the active network (decayed plage) variability is for the long-term variability for some UV wavelengths relative to the proxy’s response (contrast) to active network contributions (Woods *et al.*^2000^).

The other modeling method is considered semi-empirical or physics-based models. In these models, solar images are used to identify features on the Sun contributing to variations, then spectra are assigned for each feature type and radiative transfer based on solar heliocentric position (center-to-limb corrections), and finally the sum of all these radiance spectra provide the irradiance estimate. The radiance spectra can be measurements or physics-based spectral models. For example, the Solar Radiation Physical Model (SRPM) identifies seven different solar features and their location on the solar disk from solar visible and UV images and uses a set of physics-based spectral models for each feature type (Fontenla *et al.*, 1999, 2011, 2014). The NRLEUV model is similar type model for the EUV range and uses Ca ii K-line solar images as its input and emission measure technique for the spectra (Warren *et al.*^1996^). The Spectral and Total Irradiance Reconstruction (SATIRE) model is another good example of this modeling method (Ball *et al.*^2014^; Krivova, Solanki, and Unruh 2011; Wenzler *et al.*^2004^; Fligge, Solanki, and Unruh 2000). In the SATIRE model, the input for the variability is four components identified from solar images of the magnetic field (magnetograms), which is ultimately the source of the irradiance variability.

The variability input for both modeling methods is primarily based on a daily input, either daily proxies or daily solar images. A long-term variability input is used for some two-component proxy models, such as the 81-day average of a proxy to represent smoothing over three 27-day solar rotations. The verification of solar irradiance models is often based on comparisons to irradiance measurements with focus on daily, 27-day solar rotation, and 11-year solar cycle variations.

## Modeling Energy Variability

The modeling method presented here is empirical modeling with deficit (dark) and excess (bright) components and a focus on 6-month intervals as that time period represents the typical lifetime of each new active region. The premise is that if one can accurately determine irradiance variation over the evolution of a single active region, then solar cycle variation can be estimated as the combination of many active regions. It is well established that the SSI variability is dominated by magnetic-driven active regions (*e.g.*, Skumanich *et al.*^1984^; Lean 1997; Fontenla *et al.*^1999^; Worden, White, and Woods 1998; Worden *et al.*^1999^). More recently, Preminger and Walton (2005) have shown that the influence of individual active regions on total solar irradiance (TSI) is about seven months. Instead of examining the 27-day solar rotation variations as has been done numerous times, our new approach is to examine the total energy over 6 months as a more likely indicator for solar cycle variation than the 27-day rotation variation. Because many active regions can be on the Sun at the same time, isolating the effect of a single active region requires special analysis. One approach, as done by Preminger and Walton (2005), is to define an impulse response function (IRF) that describes the irradiance variation for a typical active region and to adjust the IRF until its convolution with multiple active regions, as identified from solar images, fits the irradiance time series. Another approach is to identify an epoch when a single active region dominates the irradiance time series.

**Figure 1:**
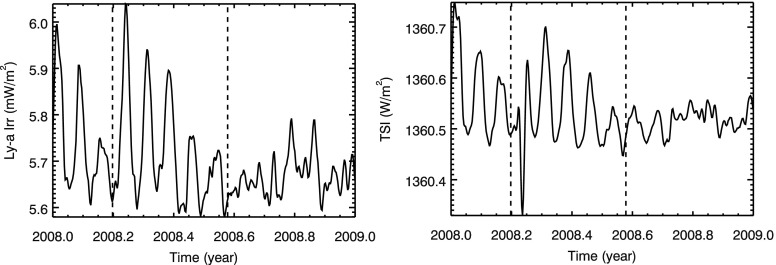
Solar Lyman-$\upalpha$ (left) and TSI (right) time series in 2008 show outburst behavior (impulse response) of a single dominant active region between the two dashed lines.

Early 2008 is the only time during the SORCE mission when a single active region is dominating the irradiance variation. Figure 1 shows the impact of the 2008 single active region for the H I Lyman-$\upalpha$ emission at 121.6 nm and the total solar irradiance (TSI). As is typical for solar UV emissions shorter than 250 nm, the Lyman-$\upalpha$ irradiance has a strong 27-day rotation peak when the active region first appears and then the 27-day rotation peaks decrease with each rotation until the active region impact is hard to detect after five solar rotations (${\sim}\,5$ months). For the TSI, the first solar rotation variation is highlighted with a strong decrease (valley) when the sunspot is near disk center but has two bright wings when the sunspot is near the limb where the surrounding faculae are brighter (Chapman *et al.*^1992^). Then for the subsequent solar rotations, the TSI variation indicates only bright contributions and is in-phase with the Lyman-$\upalpha$ variation. This TSI time series between the dashed lines in Figure 1 is very similar to the impulse response function derived by Preminger and Walton (2005).

We refer to this single active region emergence and decay affecting the irradiance as an outburst period. If one picks a day in the TSI time series for the first rotation after the new region appears, one could find a decrease of the TSI relative to the irradiance prior to the active region emergence. But it is clear that there are many more days above the pre-emergent TSI level, so there is an expectation that a single active region will have an overall positive energy release. To obtain the energy (J), one simply needs to integrate over the TSI time series (W), corrected for its starting irradiance background level. For the 2008 outburst case between the vertical dashed lines in Figure 1, one gets a positive contribution of energy from this active region, indicating that the bright faculae are more dominant than the dark sunspot. Because solar cycle variation is the consequence of multiple active regions, and because all active regions appear to have similar behavior (*e.g*., Preminger and Walton 2005; Worden, White, and Woods 1998), the TSI solar cycle variation is expected to be positive (in-phase), as is the case for the TSI record. In order for the solar cycle variation to be negative (out-of-phase), the dark sunspot component would have to be more dominant than the bright faculae, so that the irradiance integrated over time would produce a negative decrease in energy output.

While deriving SSI variations from a single 27-day solar rotation might be appropriate for ultraviolet (UV) emissions, such as for Lyman-$\upalpha$, the competing effects of the dark sunspots and bright faculae in NUV–Vis–NIR and TSI require examining the SSI variations over many months as illustrated for the TSI energy in the 2008 outburst case shown in Figure 1. This is our motivation to examine not daily or 27-day variability, but instead the energy of the irradiance over 6-month periods in the new modeling presented here. The active region impulse response result also provides guidance that estimating solar cycle variability directly from measurements should use 6-month (or longer) averages during cycle maximum and minimum periods or else selection of specific days could bias the estimate of the solar cycle variability. Because of the concern with long-term degradation trends being fully corrected over many years, our new modeling approach examines 6-month periods for variability, and we suggest that the relative energy, being the irradiance variability integrated over the 6-month period, could be representative of the spectral variations for the solar cycle.

A potential caveat to this approach is that analysis of solar cycle length records, such as active network and plage area decay as observed in solar images and the solar 10.7 cm radio flux (F10.7) (Tapping *et al.*^2007^), indicate long-term variations on the order of 600 – 800 days. Thus, the 6-month analysis window selected for this study only samples about one quarter of the long-term variations associated with solar cycle processes. Thus potential solar evolution processes with longer time constants than 6 months might not have adequate representation in the 6-month analysis results. Nonetheless, this analysis could lead to a better understanding of the role of magnetically driven active regions over the course of the solar cycle.

For studying the moderate-term (6 months) variations, we define the energy ($E$) in Equation (1a) as the integration of the variability over a 6-month period. The variability ($V$) is defined as the difference between the daily irradiance ($I$) and its minimum irradiance ($I_{\min}$). The SSI is in units of $\mbox{W}/\mbox{m}^{2}/\mbox{nm}$, making the energy units $\mbox{J}/\mbox{m}^{2}/\mbox{nm}$. For comparison of the energy to other traditional irradiance variability measurements, the relative energy ($E_{R}$) is defined as a unitless quantity as given in Equation (1b):

1a $$\begin{aligned} E =&\int V\,\mathrm{d}t\quad V=I-I_{\min} \end{aligned}$$

1b $$\begin{aligned} E_{R} =&\biggl(\int V_{R}\,\mathrm{d}t\biggr)\Bigl/t_{\mathrm{days}} \quad V_{R}=(I-I_{\min})/I_{\min}. \end{aligned}$$

While one can calculate the energy variability directly with measurements using Equation (1a)–(1b), it is more insightful to separate the variability into contributions from bright faculae and dark sunspots. The approach is to model the irradiance measurements over a 6-month period using linear regression in the form of Equation (2a) with two components: a facular excess representing the positive (in-phase) contribution and a sunspot deficit representing the negative (out-of-phase) contribution. The fitted contrasts, $C_{E}$ and $C_{D}$, are dependent on wavelength and are set to zero if the linear fit result for the contrast is not a positive number. The proxies, $P_{E}$ and $P_{D}$, are positive and negative variability time series, independent of wavelength for the excess and deficit components, respectively. The $C_{0}$ is a constant as part of the linear regression fit and is expected to be near zero if one assumes that the excess and deficit contributions are the only components needed to model the irradiance variability. In order to eliminate the possible systematic offset associated with minimum irradiance used in Equation (1a)–(1b), the constant $C_{0}$ is omitted in calculating the relative energy result as given by Equation (2b):

2a $$\begin{aligned} V_{R} =&C_{0}+C_{E} P_{E}+C_{D} P_{D} \end{aligned}$$

2b $$\begin{aligned} E_{R} =&\biggl(\int C_{E} P_{E}\,\mathrm{d}t+ \int C_{D} P_{D}\,\mathrm{d}t\biggr)\Bigl/t_{\mathrm{days}}. \end{aligned}$$

For Equation (2a)–(2b), the excess component, $C_{E} P_{E}$, is always positive (in-phase) and the deficit component, $C_{D} P_{D}$, is always negative (out-of-phase). Therefore, the irradiance is expected to have in-phase solar cycle variability if the magnitude of the excess component integral in the relative energy equation is larger than the deficit component integral. *Vice versa*, the irradiance is expected to have out-of-phase solar cycle variability if the magnitude of the deficit integral is larger than the excess integral.

The uncertainty for the energy relative variability, $\sigma_{E_{R}}$, is the root-mean-square of the four model components uncertainties as given by Equation (3a):

3a $$\begin{aligned} \sigma_{E_{R}} =&\sqrt{\sigma^{2}_{C_{E}}+ \sigma^{2}_{C_{D}}+ \sigma^{2}_{P_{E}}+ \sigma^{2}_{P_{D}}} \end{aligned}$$

3b $$\begin{aligned} \sigma_{C_{E}} =&\biggl( \int\delta_{C_{E}}P_{E}\, \mathrm{d}t\biggr)\Bigl/t_{\mathrm{days}}\qquad \sigma_{C_{P}}= \biggl(\int \delta_{C_{D}}P_{D}\,\mathrm{d}t\biggr)\Bigl/t_{\mathrm{days}} \end{aligned}$$

3c $$\begin{aligned} \sigma_{P_{E}} =&C_{E}\delta_{P_{E}}\qquad \sigma_{P_{D}}=C_{D}\delta_{P_{D}}. \end{aligned}$$

The contrast uncertainties in Equation (3b) use the uncertainties provided by the linear regression fit procedure ($\delta_{C_{E}}$ and $\delta_{C_{D}}$). The proxy uncertainties in Equation (3c) use the standard deviation of the proxies minus a 3-day smooth of the proxies ($\delta_{P_{E}}$ and $\delta_{P_{D}}$), and this represents reasonably well the day-to-day noise for the proxies. This uncertainty estimate, which is typically about 10 % of the energy variability, is considered a lower bound of the model fit uncertainty because Equation (3a)–(3c) does not include any uncertainties for the irradiance data.

The model fit over a 6-month observation period provides fitted coefficients, $C_{0}$, $C_{E}$, and $C_{D}$, and energy variability results of the excess, deficit, and total relative energy, and Equation (3a)–(3c) provides the uncertainty for a single fit. For each wavelength, the approach is to fit 6-month intervals with a time step of 2 months over the mission to provide a set of fitted coefficients and energy results over the mission. Ideally, these wavelength-dependent coefficients are constant values over time. But in reality the coefficients have variations over time as related to the noise of the irradiance measurements and also in the proxy time series. For the mission-long model fits, the average (median) of the model results is shown here and the uncertainty of model fits is estimated using the standard deviation of the contrasts fitted for the 6-month intervals over the mission. Because the model fits during cycle minima are more uncertain because of lack of solar variability to fit during low solar activity, the contrasts selected for averaging and doing this uncertainty estimate are limited to the model fit results during moderate and high solar activity. This uncertainty estimated using the multiple fits over the full mission is considered more accurate than the uncertainty estimated for a single fit as represented by Equation (3a)–(3c). Examples of this model fit approach are shown in the next section after the proxies are introduced.

## Excess and Deficit Proxies

**Figure 2:**
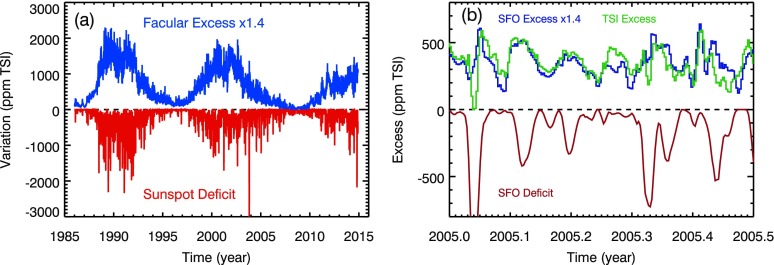
The SFO facular excess and sunspot deficit proxies in panel (a) are used in modeling the SSI variations. The alternate TSI excess proxy shown in panel (b), being the TSI minus deficit proxy, is used for modeling the photospheric emissions. The deficit proxy in panel (b) goes down to a valley of ${-}\,1200~\mbox{ppm}$ TSI near 2005.05.

The proxies selected for this analysis are the SFO facular excess derived from solar Ca ii K images at 393.4 nm and the SFO sunspot deficit derived from solar red images at 672.3 nm as shown in Figure 2a. These SFO proxies are in units of ppm of TSI as they were defined in modeling the TSI variations (Chapman, Cookson, and Preminger 2012). As will be shown later, the SFO facular excess is the only component needed for modeling the SSI UV variability for wavelengths shorter than 250 nm. For longer wavelengths, both components are needed. In modeling the SSI NUV (300 – 400 nm) with the SFO proxies, there is poor correlation (only about 0.4) regardless of which SSI data set is used. In examining the NUV fits, it is clear that the NUV facular-related peaks after a dark sunspot can be one–three days later than the SFO excess (Ca ii K) peaks. The time shift difference is likely related to the differences in center-to-limb effects between Ca ii and other wavelengths. A solution found to get better fits for the NUV is to use a different excess proxy that we refer to as the TSI excess. This TSI excess proxy, also in units of ppm of TSI, is derived as the TSI measured by the *Total Irradiance Monitor* (TIM) on SORCE minus the SFO deficit proxy. A comparison of the TSI Excess proxy to the SFO Excess proxy is shown in Figure 2b for early 2005. The TSI excess is similar in behavior to that of the SFO excess, but there are some subtle shifts when it peaks during a solar rotation. In other words, the TSI excess is more appropriate for modeling the NUV–Vis–NIR photospheric emissions than the SFO excess proxy derived from a chromospheric emission. It is also important to note that the TSI excess is a factor of 1.4 times greater than the SFO excess. Because of this difference, we use the SFO excess proxy times 1.4 for modeling the SSI UV variations so that the magnitudes of the TSI excess and SFO excess proxies used in our models are the same. The choice of which excess proxy to use is based on whether or not the emission needs a deficit component. For wavelengths with no deficit component, we assume this to be a chromospheric, transition region, or coronal emission, and we use the SFO excess proxy. For wavelengths that need the deficit component, we assume this is a photospheric emission, and we use the TSI excess proxy. This approach improved the fit correlation by about a factor of 2 for the NUV variability and also provided small improvements for the Vis–NIR.

The irradiance data sets include daily averaged values, so the gaps in the ground-based SFO measurements are filled for daily time coverage. Gaps less than three days (9 % of the record) are filled with linear interpolation of the proxy. Larger gaps in the excess proxy (38 % of the record) are filled using the 10.7 cm radio flux (F10.7) relationship in Equation (4a), and larger gaps in the deficit proxy (35 % of the record) are filled using TSI minus the filled excess proxy as given in Equation (4b):

4a $$\begin{aligned} P_{E\mbox{-}\mathrm{fill}} =&-1312.+164.\times P10\quad P10=\sqrt{(F10.7 +F10.7_{81\mbox{-}\mathrm{day}\mbox{-}\mathrm{smooth}})/2} \end{aligned}$$

4b $$\begin{aligned} P_{D\mbox{-}\mathrm{fill}} =&-28.5+ 0.750\times T\quad T=(\mathit{TSI}- \mathit{TSI}_{\min}) \bigl(10^{6}/\mathit{TSI}_{\min} \bigr)-P_{E}. \end{aligned}$$

The $P10$ definition for the F10.7 is based on prior empirical modeling of the solar ultraviolet variations with F10.7 (Woods *et al.*^2000^; Richards, Fennelly, and Torr 1994). The time series of these proxies are shown in Figure 2. The day-to-day noise, in units of ppm TSI and estimated as the standard deviation of the daily value minus the 5-day smooth, is 40 and 33 for the excess and deficit, respectively. The relationships in Equation (4a)–(4b) could be used to extend these SFO proxies back in time before the SFO measurements began in 1985.

Any 6-month period can be used to obtain fits for the contrasts, $C_{E}$ and $C_{D}$. The moderate-term (months) model fits are considered more accurate than fitting the entire mission time series because of larger uncertainties of instrument degradation trends over the long-term (years). Such an analysis of fitting every 6-month period was done first with the composite Lyman-$\upalpha$ and TSI from 1985 to 2014, and the results indicate that the constant $C_{0}$ has a solar cycle dependence if the unmodified SFO proxies are used. But by using a factor of 1.4 times the SFO facular excess, the fitted $C_{0}$ then oscillates about zero without an obvious solar cycle variation. This result implies that either a third long-term variability component is needed or that the SFO excess proxy needs a systematic scaling. As the TSI excess proxy also indicates that the SFO excess proxy needs to be scaled (see Figure 2b), we conclude that a third variability component is not required. Therefore, the SFO facular excess times 1.4 is used in the remaining part of this analysis for UV variations and it was also used in Equation (4b).

**Figure 3:**
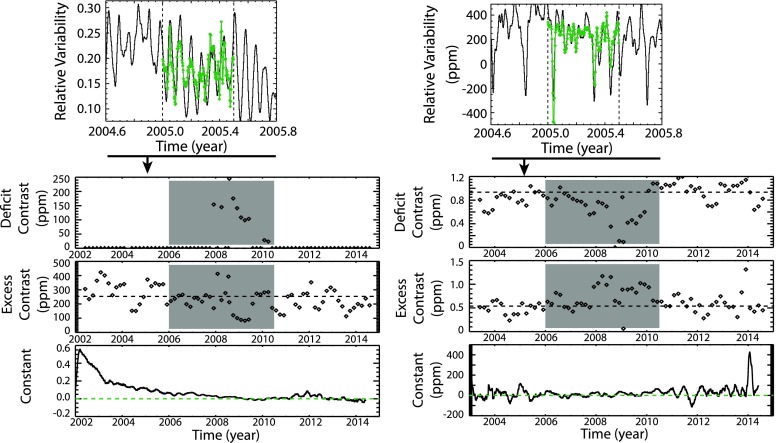
The model fits (green lines) during the 2005 outburst period between the vertical dashed lines are shown for Lyman-$\upalpha$ (left) and TSI (right). These variations are relative to the minimum irradiance in September 2008. The model fit results of the deficit contrast, excess contrast, and constant are shown in the bottom with the TIMED SEE Lyman-$\upalpha$ on the left and the SORCE TSI on the right. The average for the contrast values (dashed lines) exclude the time near solar cycle minimum (gray shaded area). The constant value is expected to be near zero; the constant deviations for Lyman-$\upalpha$ in 2002 is thought to be uncorrected degradation trend for the early phase of the TIMED mission. The constant is unitless but can be converted to irradiance units by multiplying it by the solar cycle minimum irradiance value.

Figure 3 shows example model fits for the Lyman-$\upalpha$ and TSI with a single 6-month period shown in the top panels, and the fitted contrast values over the mission are shown in the bottom panels of Figure 3. The model fits are done with 6-month periods spaced every 2 months, and the median of the contrasts during moderate to high solar activity is chosen as the contrast average. The solar cycle minimum has little variability and yielded larger fit uncertainties (gray shaded areas in Figure 3), and so the minimum period is excluded in the model average. For Lyman-$\upalpha$, the linear regression fit resulted in a deficit contrast value of zero and so all of the Lyman-$\upalpha$ variability can be explained with the positive (in-phase) excess component. For TSI, the magnitude of the excess and deficit components are similar but with the excess component slightly larger than the deficit. Therefore, the addition of the positive excess and negative deficit components predicts a small positive (in-phase) solar cycle variation, as is expected for TSI.

## SSI Data Sets

SORCE has two SSI instruments that cover the spectral range from 115 nm to 2400 nm. The *Solar Stellar Irradiance Comparison Experiment* (SOLSTICE) is a grating spectrometer for the wavelength range of 115 nm to 310 nm with 0.1 nm spectral resolution and with 10 – 15 measurements per day (McClintock, Rottman, and Woods 2005; Snow *et al.*^2005^). The *Solar Irradiance Monitor* (SIM) is a prism spectrometer for the wavelength range of 240 nm to 2400 nm with low spectral resolution (0.6 nm at 240 nm and 24.6 nm at 2200 nm) and with one–two measurements per day (Harder *et al.*^2005^). The SIM data from its photodiodes in the 250 nm to 1600 nm range are included in these analyses. Both SOLSTICE and SIM had recent updates in late 2014 to improve their degradation corrections, and these current versions are used in this analysis (SOLSTICE version 14 and SIM version 22).

Three other solar irradiance measurements are also presented using the energy modeling technique. The *Solar Extreme ultraviolet Experiment* (SEE) aboard the NASA *Thermosphere, Ionosphere, Mesosphere, Energetics and Dynamics* (TIMED) satellite is a grating spectrograph for 27 nm to 190 nm with 0.4 nm resolution and soft X-ray (SXR) filter photometers for broad band measurements in the 0.1 nm to 27 nm range (Woods *et al.*^2005^). The TIMED SEE version 11 data products are used here. The *Solar Ultraviolet Spectral Irradiance Monitor* (SUSIM) aboard the NASA *Upper Atmosphere Research Satellite* (UARS) is a grating spectrometer for 115 nm to 410 nm (Brueckner *et al.*^1993^), and UARS SUSIM version 22 data are used here. The *Solar Backscatter UltraViolet* (SBUV) instrument measurements provide solar UV measurements in the 200 nm to 400 nm range from 1978 to the present from several different NOAA satellites. The composite solar UV irradiance compiled by DeLand and Cebula (2008) is used in this analysis as the means to study NOAA-11 SBUV daily solar irradiance variability during Solar Cycle 22.

The measurements over the full mission range have been fit in 6-month intervals every 2 months. Additionally, specific months are used for estimating solar cycle variations directly from the measurements. The dates for the solar variability measurements are listed in Table 1 for the different missions. The SORCE mission started after Solar Cycle 23 (SC-23) maximum, so its SC-23 “maximum” in April 2004 represents variability that is about half as much as expected for the true Solar Cycle 23 maximum. The solar rotation variability can have large negative variations in the NUV–Vis–NIR ranges when dark sunspots dominate and thus are not expected to compare well to solar cycle variability nor to these new energy modeling results; nonetheless, the 27-day rotation variability is shown in the comparisons for clarifying the spectral differences between short-term and longer-term variations.

**Table 1:** Dates used for solar variability comparisons. The solar cycle (SC) variations use a 180-day average centered on the listed date, and 27-day solar rotation (Rot) variations use a 3-day average centered on the listed date. The format of the dates is year and day of year (DOY).

Mission/Instrument	Solar Cycle	SC Min	SC Max	Rot Min	Rot Max
NOAA-11/SBUV	22–rise	1986/320	1989/360	1989/349	1989/362
UARS/SUSIM	22–decline	1996/087	1992/034	N/A	N/A
UARS/SUSIM	23–decline	1996/087	2004/111	2005/010	2005/017
TIMED/SEE	23–decline	2008/259	2002/231	2005/010	2005/017
TIMED/SEE	24–rise	2008/259	2011/312	N/A	N/A
SORCE	23–decline	2008/259	2004/111	2005/010	2005/017
SORCE	24–rise	2008/259	2011/312	N/A	N/A

## UV Results

The results from the energy modeling technique can be divided into the ultraviolet range (0.1 nm to 250 nm) where the excess component is the only needed component and the NUV–Vis–NIR ranges where the excess and deficit components are both needed at most wavelengths. The UV results are presented in this section, and the NUV–Vis–NIR results are presented in the following sections.

**Figure 4:**
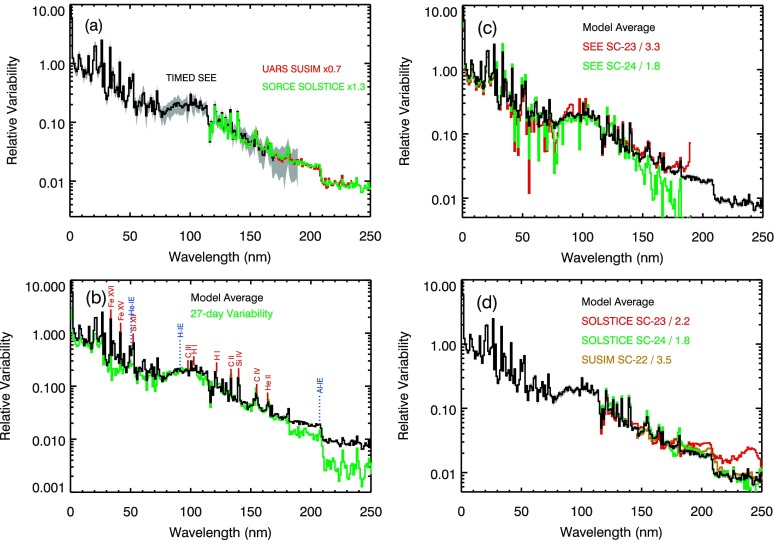
The average energy relative variability is consistent for TIMED SEE, SORCE SOLSTICE, and UARS SUSIM in the UV range as shown in panel (a). The composite of energy variability shown in panels (b), (c), and (d) includes TIMED SEE at wavelengths shorter than 115 nm and SORCE SOLSTICE at wavelengths longer than 115 nm. Panels (b), (c), and (d) compare these energy variability results to the 27-day solar rotation variability, TIMED SEE solar cycle variability, and SORCE SOLSTICE/UARS SUSIM solar cycle variability, respectively. The energy variability uncertainty (RMS of standard deviation of fitted parameters) is included as the gray region in panel (a) for SEE data.

The average relative energy results from Equation (2a)–(2b) are shown in Figure 4a at 1 nm resolution and up to 250 nm from TIMED SEE, SORCE SOLSTICE, and UARS SUSIM measurements. The model fits for this UV range yielded zero for the deficit contrast (as only positive contrast values were allowed), so only the excess component is needed for the UV range shorter than 250 nm. The spectral dependence of the model fits are very similar between the three different missions (UARS, TIMED, SORCE) as shown in Figure 4a. A scaling factor is needed for this comparison because different missions covered different levels of the solar cycle. The TIMED SEE results are plotted without any scaling. The UARS mission included the peaks of solar cycle maximum and thus cover higher levels of solar activity than TIMED, so the UARS SUSIM model fit results have a lower scaling factor of 0.7 relative to TIMED. Conversely, the SORCE mission started after the TIMED mission and did not cover any high solar activity in Cycle 23, so the SORCE results have a higher scaling factor of 1.3 relative to TIMED. Because these model fits are over three different solar cycles, the similarities in the model fit results indicate that solar variability appears to have the same spectral behavior independent of solar cycle or solar activity level.

The model fit results from each instrument are shown only if the model fit correlation is larger than 0.6. This condition consequently excludes TIMED SEE results between 170 nm and 190 nm and excludes UARS SUSIM results from 115 nm to 145 nm and from 235 nm to 410 nm. This condition also excludes SORCE SOLSTICE results from 290 nm to 310 nm, but we note that this range is outside the range of Figure 4. In these cases, their measurement precision (day-to-day noise) is too large to get reasonable model fits.

The composite energy results in Figure 4b, c, and d include 0 – 115 nm from TIMED SEE and 115 – 250 nm from SORCE SOLSTICE. The energy spectral variability results are similar to 27-day solar rotation variations as shown in Figure 4b. The 27-day solar rotation variation is chosen for when a new active region appeared in early 2005 and is calculated using the 3-day average centered on 2005/017 and 2005/010 using the TIMED SEE data (0 – 115 nm) and SORCE SOLSTICE data (115 – 310 nm). The relative variability generally increases with shorter wavelengths. The upper photosphere and lower chromosphere emissions have less variability, and these dominate primarily in the 150 nm to 250 nm range. The transition region and coronal emission lines, with several of them labeled in Figure 4b, have enhanced variability with one to two orders of magnitude more than the chromospheric variability. Some of the ionization edges (IE) are also labeled in Figure 4b. We note that the UV relative variability (from Equation (1b)) is large, but the variability in units of $\mbox{W}/\mbox{m}^{2}$ (from Equation (1a)) for the UV 0 – 250 nm range is only a minor contribution to the TSI variability.

For this comparison in Figure 4b, the rotational variation has a magnitude similar to that of the energy variation for wavelengths shorter than 180 nm. At longer UV wavelengths, the rotational variation is more than a factor of two less than the variation in energy. Because there is no deficit contribution for the UV range, the spectral dependence for the rotational variation and energy variation is expected to be similar but not identical. For example, Woods *et al.* (2000) showed for the far ultraviolet (FUV: 120 – 200 nm) that the ratio of solar cycle variation to rotational variation is larger for transition region emissions than chromosphere emissions due to enhanced brightness of active network regions in solar images for the transition region emissions than the chromosphere emissions. What is new here is the systematic difference between 180 nm and 250 nm with the ratio of the energy variation to the rotation variation stepping up to a ratio of about two at 180 nm and again to a ratio of about three at 207 nm. The step up at 207 nm is related to the Al ionization edge. The step up at 182 nm does not correspond to any ionization edge but is suspiciously near the 180 nm transition between FUV and middle ultraviolet (MUV: 200 – 300 nm) detectors for SOLSTICE. The emissions in this wavelength range are primarily from the lower chromosphere and upper photosphere. The comparison suggests that one needs to use caution in scaling 27-day solar rotation variability for estimating outburst (solar cycle) variability.

Figure 4c shows the UV energy results compared to TIMED SEE solar cycle variability measurements. The Solar Cycle 23 (SC-23) variability uses 180-day averages in August 2002 (maximum) and in September 2008 (minimum), and the Solar Cycle 24 (SC-24) variability uses November 2011 (maximum) and the same minimum in September 2008 (see Table 1 for exact dates). The solar cycle ratio (maximum to minimum) is typically a factor of 2 – 10 for the extreme ultraviolet (EUV: 0 – 120 nm) and a factor of 1.2 – 1.6 for the far ultraviolet (FUV: 120 – 200 nm). For wavelengths shorter than 150 nm, the solar cycle variability is much larger than the stability uncertainties for TIMED SEE measurements, which are about 1 – 2 % per year. Therefore, the solar cycle variations from TIMED SEE are less sensitive to instrument degradation corrections over the long term; consequently, this comparison provides validation that the energy results can be representative of the solar cycle variability. The divergence of about 1 % at longer than 170 nm between energy variability and the solar cycle variability, notably for SC-24, could be related to uncorrected degradation for TIMED SEE, but these differences are within the TIMED SEE stability uncertainty of 1 – 2 % per year.

Note that the divergence for the rotational variability and energy variability in Figure 4b cannot be related to TIMED SEE stability because the rotational variability result is from SORCE SOLSTICE for longer than 115 nm. Furthermore, the stability uncertainty contribution for the rotational variability is much smaller than solar cycle variability studies.

Because solar cycle variability includes the effects of many active regions, the solar cycle variability is scaled down for this comparison to have similar levels as the energy result in the 130 – 150 nm range. The spectral variability of the energy result is most similar to the SEE Solar Cycle 23 variability. There are, however, some notable differences for the SEE Solar Cycle 24 variability comparison. For one, the scaling factor for SC-24 variability is about two times less than the SC-23 scaling factor; thus, confirming that SC-24 activity is much weaker than in SC-23. Secondly, some of the coronal emission lines in the EUV have slightly more variability in SC-24 than either in SC-23 or the energy model result. Because the SFO facular excess is derived from chromospheric Ca ii K images, this excess proxy may not be appropriate for modeling coronal lines.

Figure 4d shows the UV energy results compared to SORCE SOLSTICE and UARS SUSIM solar cycle variability measurements. These results are also based on using 180-day averages, and the dates for SC-24 variability are the same as for TIMED SEE variability (Figure 4c). The dates for SORCE SOLSTICE SC-23 variability are September 2008 (minimum) and April 2004 (moderate level as SORCE was launched after SC-23 maximum). The dates for the UARS SUSIM SC-22 variability are March 1996 (minimum) and January 1992 (maximum). As for Figure 4c, these solar cycle variability results are scaled to match the energy variability for the 130 – 150 nm range. All three solar cycle results have very similar spectral dependence as the energy variability, thus providing additional validation that the energy variability result is appropriate for solar cycle activity. One notable difference is that the SOLSTICE SC-23 variability is higher near 240 nm; this difference is thought to be related to interpolation across just a few wavelengths in the SOLSTICE in-flight stellar calibrations during the early years of the SORCE mission.

The differences between the various solar cycle measurements and the energy variability results between 0 and 250 nm suggest that the energy variability uncertainty might be about 20 % of the variability. For example, the estimated uncertainty for the 2 % energy variability near 200 nm is ${\pm}\,0.4~\mbox{\%}$. The energy variability uncertainties plotted as the gray regions for TIMED SEE data in Figure 4a are derived from the standard deviation of the fitted contrasts and are about 25 %. The calculated model fit uncertainties for a single fit from Equation (3a)–(3c) are about 10 % of the energy variability and thus appear to be about a factor two lower than the uncertainties estimated by the fitted contrast standard deviation and by the differences between the different solar cycle variability measurements. This difference in uncertainties may reflect that the uncertainty of the constant may be needed in Equation (3a)–(3c) and/or that the relationship of the energy variability to the proxies can change over the solar cycle. The larger uncertainty as derived from the standard deviation of the fits over the full mission is adopted for all of the energy variability plots.

This comparison of different solar cycle variability is also important as it indicates similar spectral dependence over the past three solar cycles. The SOLSTICE SC-23 scaling factor (2.2) is much less than the SEE SC-23 factor (3.3) because of different dates chosen at different activity levels. There is the expectation from various solar activity proxies that SC-22 maximum and SC-23 maximum should be similar, and this is confirmed with both SEE SC-23 scaling factor and SUSIM SC-22 factor being a factor of about 3.4 relative to the energy variability.

## NUV Results

**Figure 5:**
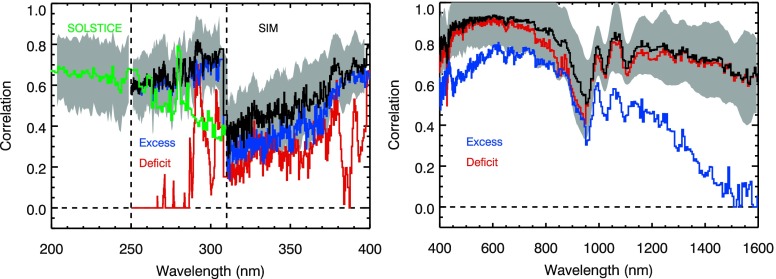
The combined correlation for the energy model fit is averaged over the SORCE mission and is shown as the black and green lines for SIM and SOLSTICE, respectively. The mission-average correlation results of the individual excess and deficit components are also shown as the blue and red lines, respectively. The gray region includes the standard deviation for the combined correlation results.

Because of the wide spectral coverage for the NUV–Vis–NIR ranges, those results from SORCE will be shown in two spectral ranges: 200 – 400 nm and 400 – 1600 nm. But first, a deficiency in the proxy model is discussed for the NUV (300 – 400 nm) range as shown by low correlation values in Figure 5. The excess component is most important for the UV range shorter than 270 nm. The deficit component is most important for the Vis–NIR ranges (${>}\,400~\mbox{nm}$). The NUV fits were significantly improved in using the TSI excess proxy instead of the SFO excess proxy, but some NUV wavelengths still have low correlation (${<}\,0.6$) associated with higher day-to-day noise for those SIM measurements. In the previous section, the energy model results with correlation of less than 0.6 were excluded in the UV result plots because they were not considered accurate enough due to the measurements having high day-to-day noise affecting the model fits. The wavelengths with low correlation (${<}\,0.6$) in Figure 5 are perhaps also associated with SORCE measurements with higher day-to-day detector noise, but there also appears to be reduced model correlation when both excess and deficit components are important. The SORCE SOLSTICE has low signals at longer than 290 nm. The 310 – 365 nm range for the SIM visible (Vis1) photodiode has signal-to-noise ratio (SNR) less than 1000 and drops to about 230 at 310 nm, thus making precision somewhat marginal for a study of this kind. In the 900 – 950 nm range Vis1 photodiode has residual temperature noise and also produces lower stability in this wavelength range. Ideally, we would exclude the poorer fit results, but we show all of the SIM results here to provide full spectral coverage. The energy model was fit to the UARS SUSIM data that also overlaps with the SORCE mission period, and the SUSIM model correlation is also low (${<}\,0.6$) in both the 115 – 145 nm and the 235 – 410 nm ranges and is slightly lower than SIM correlations in the 300 – 400 nm range. Similarly, the energy model was fitted to the NOAA-11 SBUV data. The SUSIM and SBUV model results are comparable to the SIM model results as discussed later in this section.

**Figure 6:**
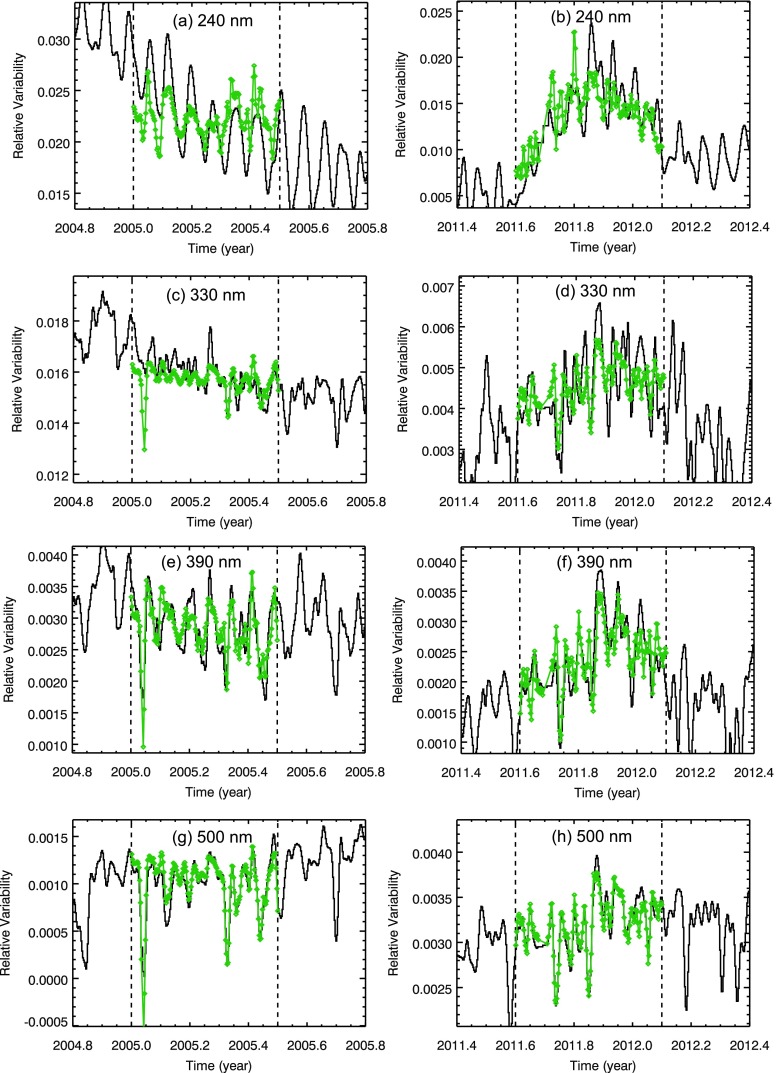
Example model fits (green lines) are shown for 6-month periods in 2005 (SC-23) and 2011 (SC-24) for SORCE SOLSTICE 230 – 250 nm (a, b), SIM 320 – 340 nm (c, d), SIM 380 – 400 nm (e, f), and SIM 490 – 510 nm (g, h). The observations are the black lines. The relative measurement precision (noise) for the SORCE measurements are estimated to be 400 ppm, 300 ppm, 200 ppm, and 100 ppm for 240 nm, 330 nm, 390 nm, and 500 nm, respectively, and this noise is less than a vertical tick mark in each plot. The dashed lines indicate the fit range for the 6-month period.

With some NUV wavelengths having poorer correlation than other wavelength ranges, a few examples of the model fits are shown in Figure 6. The model fit for the SOLSTICE 230 – 250 nm irradiance has only an excess component, and the model fit for the SIM 490 – 510 nm irradiance is dominated by the deficit component. In cases where only one component is primarily needed, the fits are significantly better (correlation ${>}\,0.8$). The NUV example fits in Figures 6c–6f need both deficit and excess components at some wavelengths, and the NUV range is where the model fits have the lowest correlation. Despite the lower confidence for the NUV model fits, we have included them in the figures for completeness of showing results at all wavelengths shorter than 1600 nm.

**Figure 7:**
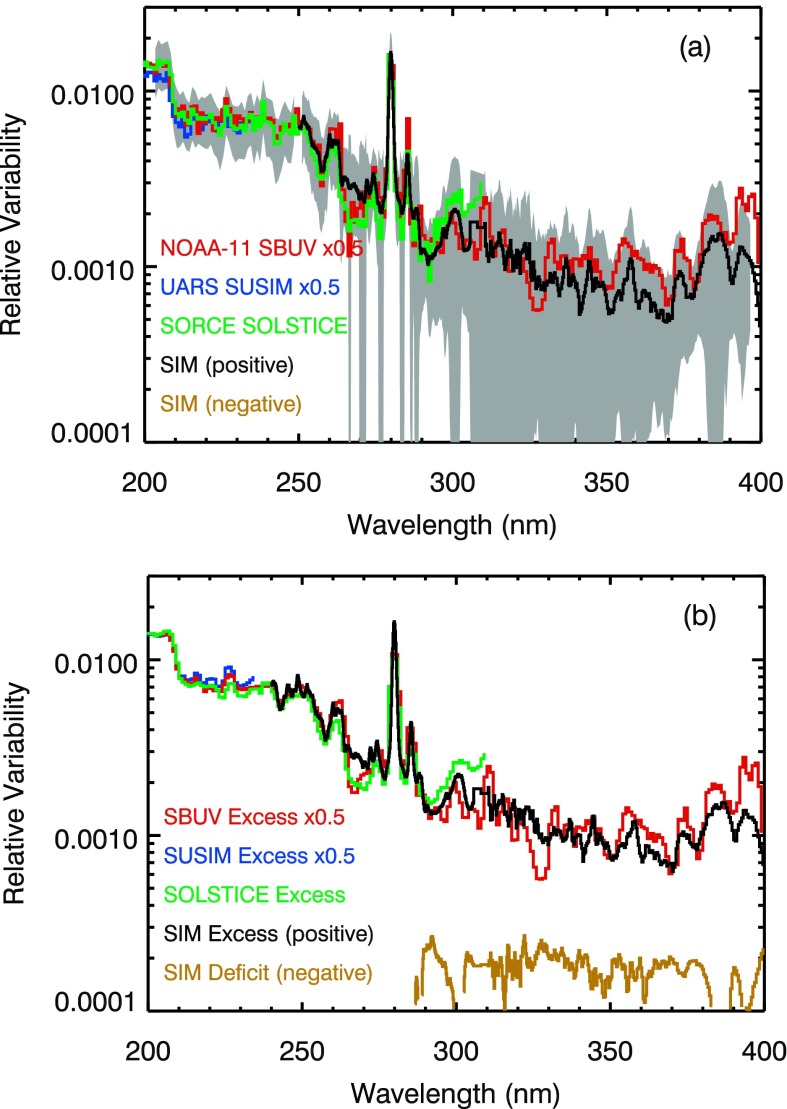
The average energy relative variability is consistent for SORCE SOLSTICE, SORCE SIM, UARS SUSIM, and NOAA-11 SBUV in the MUV and NUV ranges, as shown in panel (a). The excess contributions for these energy variability results are given in panel (b). The SIM deficit contributions are also shown in panel (b). The others’ deficit and negative contributions are not shown as they are small and below the scale. The energy variability uncertainty (RMS of the standard deviation of the fitted parameters) is included as the gray region in panel (a) for SORCE data.

**Figure 8:**
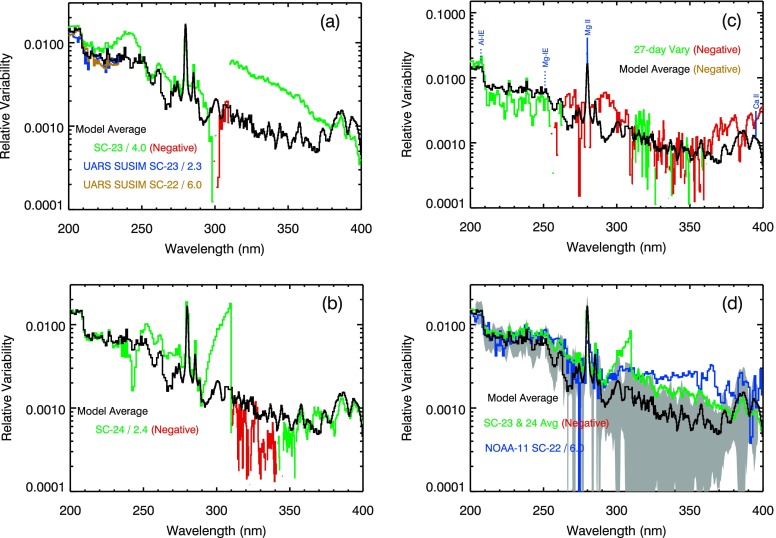
The composite of energy variability includes SORCE SOLSTICE between 200 and 270 nm and SORCE SIM between 270 nm and 400 nm, and this energy variability is compared to (a) SORCE SC-23 variability (green/red) and UARS SUSIM SC variability (blue and gold), (b) SORCE SC-24 variability (green/red), (c) SORCE 27-day solar rotation variability (green/red), and (d) average of SORCE SC-23 and SC-24 variability results (green/red) and NOAA-11 SBUV SC-22 variability (blue). The SORCE measured variability results are shown in green for positive (in-phase) and in red for negative (out-of-phase) cases. The energy variability uncertainty (RMS of standard deviation of fitted parameters) is included as the gray regions in panel (d).

The average relative energy results in the 200 nm to 400 nm range are shown in Figure 7, and Figure 8 compares those results to solar cycle measurements from SORCE SOLSTICE, SORCE SIM, UARS SUSIM, and SBUV. The energy variability results shown in Figure 7 only have positive values, but the deficit component does have weak contributions in the 290 – 400 nm range but are off scale in Figure 7a. The energy variability results from four different instruments have a very similar spectral dependence as shown in Figure 7a. The differences are larger at wavelengths longer than 290 nm partly because the day-to-day detector noise is of the order of the energy variability (0.1 %). The SORCE and UARS missions do have some overlap, but the NOAA-11 SBUV results are from an earlier time period. One of the differences in this comparison is that SORCE SOLSTICE results deviate from the others in the 290 nm to 310 nm range, and this difference is the result of poor model fits for SOLSTICE in this wavelength range (model fit correlation being less than 0.6). The UARS SUSIM results in Figures 7 and 8 are only shown for the 145 – 235 nm range due to the low correlation at its other wavelengths. The other notable difference in the energy variation comparison (Figure 7a) is that the SBUV energy variation is slightly higher than the SIM variation between 350 nm and 400 nm. As noted above, there is a higher uncertainty for the energy variation results in the NUV because the model fit correlation is low for all of the different NUV measurements. Nonetheless, there is reasonable agreement in the spectral dependence in the NUV energy variability between SIM and SBUV. Those NUV differences of about 50 % are fairly consistent with the uncertainties of the model fits that are shown as the gray regions in Figure 7a.

The SIM excess and deficit contributions are shown in Figure 7b. Note that the total energy variability shown in Figure 7a is the sum of the positive excess and negative deficit contributions. The excess contribution dominates in the UV, so the excess contribution variability looks much like the energy variability (positive excess plus negative deficit). The excess contributions from SOLSTICE, SUSIM, and SBUV are also shown in Figure 7b, but their deficit contributions, being small, are omitted for clarity.

The energy variability in Figure 8 generally increases with shorter wavelengths as it did in the FUV. The composite energy results in Figure 8 include 115 – 270 nm from SORCE SOLSTICE and 270 – 400 nm from SORCE SIM. The 27-day rotation variability shown in Figure 8c for the specific period in 2005 has both positive (excess brightening) and negative (deficit darkening) variations. There is very little resemblance of the 27-day solar rotation variability to the energy variability in this comparison, except for the good agreement at a few wavelengths like the Mg ii emission at 280 nm. The 27-day solar rotation variation is for the same period used in Figure 4 for TIMED SEE comparison. It is clear that this 27-day solar rotation variability with positive and negative variations cannot be scaled in a linear way to agree with the energy variability that only has positive variations. For wavelengths longer than 250 nm that have competing effects of dark sunspots and bright faculae, it is not very useful to compare the short-term 27-day solar rotation variability to the longer-term variations from the energy model or measurements of the solar cycle variability.

The measured solar cycle variations from Solar Cycles 22 – 24 are compared to the energy variation in Figures 8a, 8b, and 8d. The Solar Cycle 22, 23, and 24 variations used the same dates as defined for Figure 4d and listed in Table 1. The data set scaling factors are different between Figures 4 and 8 because the energy variability results in Figure 4 are the average during the SEE mission that has higher Solar Cycle 23 activity than the SORCE mission whose data are used for Figure 8. The NOAA-11 SC-22 variations during the rising phase of SC-22 with maximum in December 1989 and minimum in November 1986 (see Table 1 for exact dates) are also included in Figure 8. The SC-22 rising phase (NOAA-11) and SC-22 declining phase (UARS SUSIM) results have the same scaling factor of 6.0. The SORCE SC-23 and SC-24 results used the standard SORCE SSI Level 3 product where SOLSTICE data are used at shorter than 310 nm and SIM data at longer than 310 nm. The SOLSTICE SC results are not as reliable in the 290 – 310 nm range due its low signals in that range. The solar cycle variations are mostly consistent with the energy variability up to the Mg i ionization edge near 250 nm and for the Mg ii emission at 280 nm. The uncertainties for the MUV and NUV energy relative variability results are estimated by the model fits to be about 25 % at shorter than 286 nm and larger than 50 % for 286 – 400 nm range.

There is much more uncertainty in the NUV energy variability results as indicated as the larger gray regions in Figure 8d. There are significant differences between 290 nm and 400 nm for the solar cycle comparisons in Figure 8. These differences are amongst the three different measurement sets (SORCE SIM, UARS SUSIM, and NOAA-11 SBUV) and over three different solar cycles. The NUV energy variability is less than the SORCE SIM SC-23 and NOAA-11 SC-22 measurements but is larger than the SORCE SIM SC-24 variability. Both SORCE solar cycle measurements indicate negative (out-of-phase) variation in a narrow range near 300 nm; whereas, the energy variability and NOAA-11 solar cycle variability only have positive (in-phase) variation for those wavelengths. Another comparison can be made by averaging the SORCE SC-23 and SC-24 variability results as shown in Figure 8d. This solar cycle variability average on opposite sides of the same solar cycle minimum has the advantage that it could cancel out any uncorrected instrument degradation trend that is systematic (linear with time). We note that this averaged solar cycle variability does not have any out-of-phase contributions in the NUV range. However, we warn that the average of two results with large noise (uncertainty), such as in the SOLSTICE 290 – 310 nm range, could instead provide an estimate with even larger uncertainty.

One possible conclusion from the NUV comparisons is that the energy variability result could be useful as an estimate for the solar cycle variability spectral dependency. We note, however, that the model fits in the NUV had low correlation (see Figure 5) and thus larger uncertainty for the energy variability in the NUV. The spectral dependence of the energy variability in the NUV is fairly flat at about 0.1 %. The solar cycle variability comparison in Figure 8d suggests that the NUV variability could be more like 0.2 % to 0.3 %. It is unclear what the true solar cycle variability might be in the 290 – 400 nm range based on these comparisons.

As an example for SIM solar cycle variability accuracy, the SIM-A 350 nm data have a degradation trend of about ${-}\,1.9~\mbox{\%}$ per year, the degradation in SIM-A is corrected by comparisons with SIM-B to give a trend uncertainty of 0.17 %, and day-to-day noise (repeatability) of 0.13 %, so the degradation correction is noise limited. The effect of noise on the variability result can be reduced by averaging over many days, so the degradation trend uncertainty of 0.17 % is the dominant factor in SIM solar cycle variability uncertainty at 350 nm. The uncertainty at 350 nm for the energy variability result is estimated to be 0.05 %, but the spectral flatness of the energy variability at 0.1 % may indicate a limitation of the energy model to 0.1 % for the 300 – 400 nm range due to SIM precision limitation. These uncertainties can be compared to the intrinsic solar cycle variability at 350 nm, which may actually be less than 0.1 %.

Because of the consistency in solar cycle variability between these instruments at the shorter wavelengths, these NUV differences could indicate that the solar cycle variability for the NUV may not be measurable with these instruments at the required accuracy. In other words, the intrinsic solar variability could be smaller in the NUV than the uncertainties associated with instrument degradation corrections. Some of these differences might be resolved with additional studies; for example, there are on-going studies to better understand the instrument trends for the SORCE instruments and to improve their data product accuracy.

Except for confirming the UV result that SC-24 has smaller cycle maximum than SC-23, there are no clear (accurate) conclusions for the NUV variability. Until there is additional analysis, or new NUV SSI measurements, the energy variability and solar cycle variations in Figure 8d could be considered to represent the range of solar cycle variability for the NUV. Note that the variability values in Figure 8d need to be scaled up by factor of 2.4 for SC-24 true maximum and up by factor of 6.0 for SC-21, 22, and 23 true maximum.

## Vis–NIR Results

**Figure 9:**
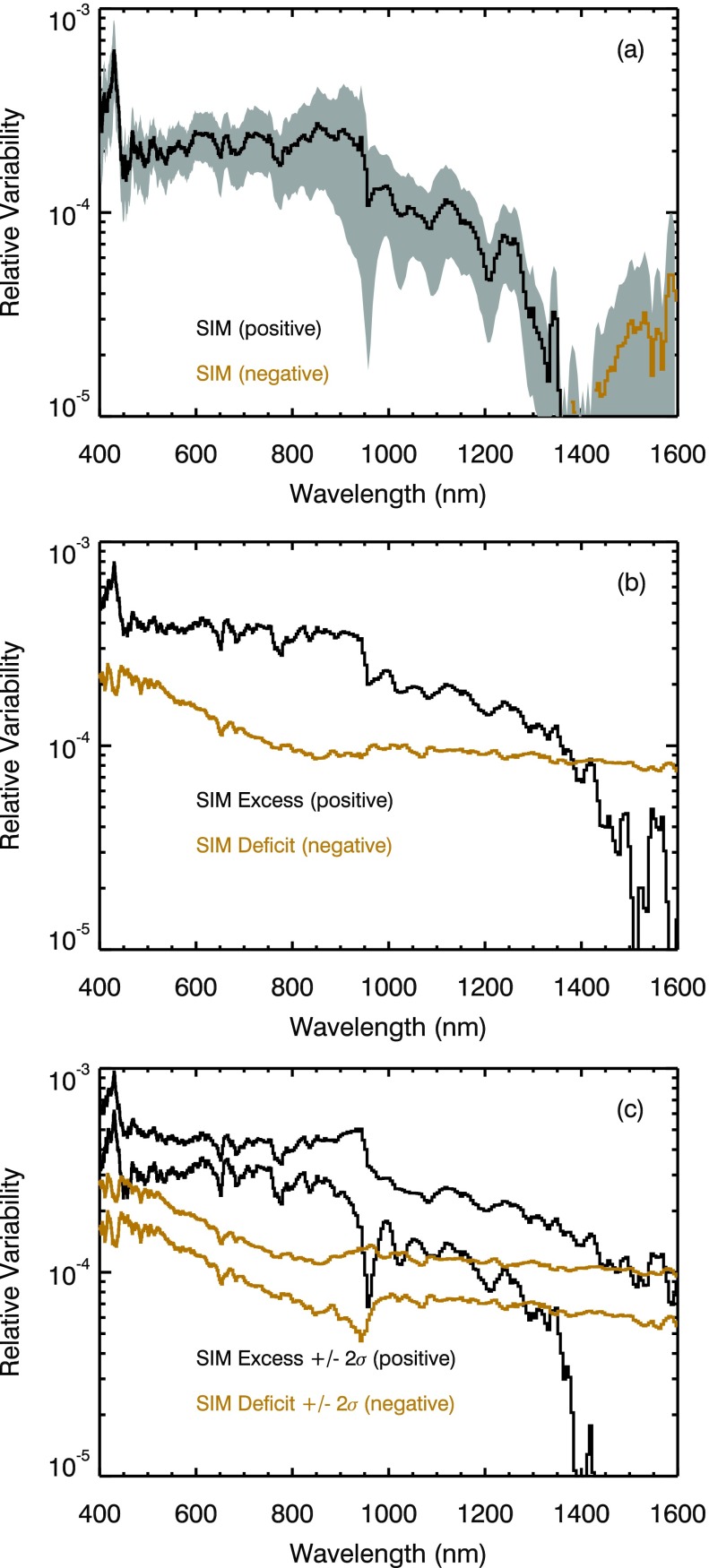
The average energy relative variability is shown in panel (a) for SORCE SIM in the Visible and NIR ranges. The SIM excess and deficit contributions for these energy variability results are given in panel (b). Unlike the UV range where excess contributions dominate, the deficit contribution is important throughout the Vis–NIR ranges. The out-of-phase (negative) variability is in the 1400 – 1600 nm range as shown in panel (a) and also indicated in panel (b) when the Deficit contribution is larger than the Excess contribution. Panel (c) shows the Excess and Deficit contributions with 2$\sigma$ uncertainties to illustrate that the out-of-phase contribution might not exist at any Vis–NIR wavelengths or might possibly extend down to 900 nm. The gray region in panel (a) includes the uncertainty for the energy variability results, being about 30 % for 400 – 900 nm and larger than 50 % for longer than 900 nm.

**Figure 10:**
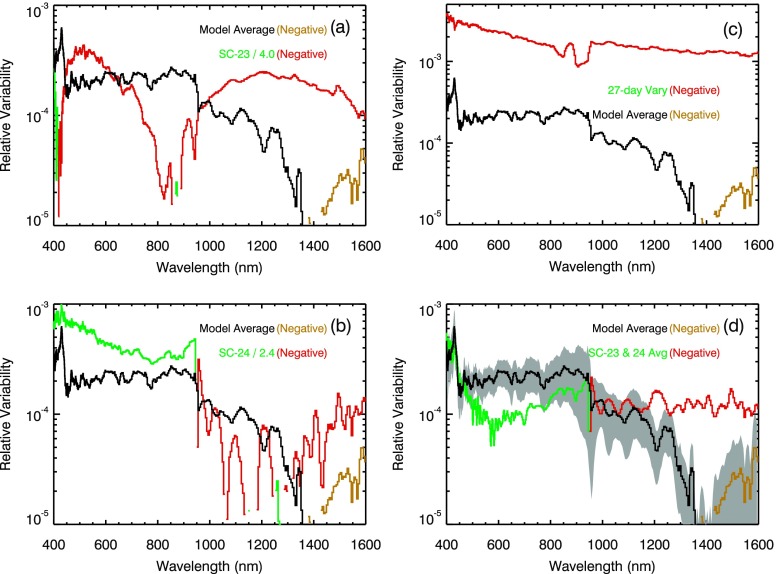
The SIM energy variability is compared to (a) SIM SC-23 variability, (b) SIM SC-24 variability, (c) SIM 27-day solar rotation variability, and (d) average of SIM SC-23 and SC-24 variability results. The SIM measured variability results are shown in green for positive (in-phase) and in red for negative (out-of-phase). The energy results are shown in black for positive and in gold for negative variability. The energy variability uncertainty (RMS of standard deviation of fitted parameters) is included as the gray regions in panel (d).

The average relative energy results in the 400 nm to 1600 nm range are shown in Figure 9, and Figure 10 compares those results to solar cycle measurements from SORCE SIM. The energy variability in these wavelengths has both positive and negative energy results with the negative energy (out-of-phase) being in the 1400 – 1600 nm range. The out-of-phase solar cycle variability in the NIR is expected because the facular regions are dark relative to the quiet Sun in the NIR (Foukal *et al.*^1990^), and this behavior is related to the $\mathrm{H}^{-}$ opacity dominating the radiation output in the NIR. In the visible, the facular regions are darker than quiet Sun near disk center but are brighter near the limb (Topka, Tarbell, and Title 1997), and this behavior is related to the viewing geometry of the cooler faculae floor near disk center and the hotter faculae wall near the limb. Although the 27-day rotation variability is reasonably well understood in the visible and NIR (*e.g.*, Pagaran *et al.*2011b), the net effect of the competing contributions over the moderate-term (6-month) period is not well established and is at the heart of the solar physics debate concerning the SIM solar cycle variability result described by Harder *et al.* (2009).

The Harder *et al.* (2009) solar cycle variability results included out-of-phase solar cycle variations in the 400 – 691 nm and 972 – 1600 nm ranges. The average energy variability result only has out-of-phase cycle variations in the 1400 – 1600 nm range. The total energy (positive excess plus negative deficit) as shown in Figure 9a is the magnitude of the total energy so that both positive (black) and negative (gold) values can be displayed with the ordinate log scale. We note that the points where the energy variability goes through zero in Figure 9a are the wavelengths with the same magnitude for the excess and deficit contributions as are shown in Figure 9b. The uncertainty for the model fit in the NIR is large at 50 %, so the Excess and Deficit contributions with 2$\sigma$ uncertainties are examined (Figure 9c) to estimate what other wavelengths might have out-of-phase contributions. Figure 9c illustrates that out-of-phase contributions might not exist at any Vis–NIR wavelengths for the case of the high excess contribution and the low deficit contribution. Figure 9c also shows that out-of-phase contributions might possibly extend down to 900 nm and near 450 nm for the case of the low excess contribution and the high deficit contribution. This difference in the spread of out-of-phase wavelengths is the consequence of the uncertainty of this energy model, but it could also be suggestive that the range of wavelengths with out-of-phase behavior could be different during different times of the solar cycle. As shown in the next section, the NRLEUV and SATIRE models have solar cycle out-of-phase contributions similar to that shown in Figure 9b.

The comparison of the energy variation to the 27-day solar rotation variability in Figure 10c indicates that all of the Vis–NIR wavelengths have a strong deficit contribution when a new sunspot appears (beginning of active region outburst). That is, the 27-day rotation variability is all negative in the Vis–NIR and is much larger than the magnitude of the energy variation. It is also possible to pick different dates for the 27-day rotation variability comparison and get all positive variations in the Vis–NIR for when the excess contribution is more dominant. As pointed out in the previous section, it is not informative to compare solar rotation variations to solar cycle variations for wavelengths with competing effects of dark sunspots and bright faculae.

The SIM Solar Cycle 23 and 24 variability results are compared to the energy results in Figures 10a and 10b. The dates and scaling factors for the solar cycle variations are the same as defined for Figure 8 (see Table 1 for specific dates). As done for Figure 8d, the average of the SIM SC-23 and SC-24 variability is plotted in Figure 10d. The Vis–NIR energy variability is only about 0.02 % and is about a factor of 5 less than the NUV relative variability. There are large differences between the SIM solar cycle results and the energy variability result in the Vis–NIR ranges, so these SIM solar cycle measurements cannot be used to validate the Vis–NIR energy variability result. The SIM SC-24 variability results are expected to have larger uncertainty than the SIM SC-23 results because SORCE operations have required power-cycling SIM off during orbit eclipse since the beginning of SC-24 (2009). This power-cycling mode introduces wider range of temperature variation for SIM, and even though temperature corrections are made as part of SIM data processing, the uncertainties for SIM irradiance results are larger in SC-24.

The magnitude of the variability is very small, so understanding accurately the instrument degradation trends is critical for studying the Vis–NIR ranges. This new approach of examining active region energy (integration of irradiance over 6 months) has potential for being more accurate than traditional solar cycle variation comparisons over many years for wavelengths where the degradation correction uncertainties are comparable in magnitude to the intrinsic solar variability. In other words, the energy result is much less sensitive to instrument degradation trending over a few months than solar cycle variability results over several years. For example, SIM-A 500 nm data have a degradation trend of ${-}\,0.17~\mbox{\%}$ per year, degradation trend uncertainty of 0.06 %, and day-to-day noise (repeatability) of 0.012 %. The effect of noise on the variability result can be reduced by averaging over many days, so the degradation trend uncertainty is the dominant factor in SIM solar cycle variability uncertainty, as was the case also in the NUV. The uncertainty of the energy model results in the Vis–NIR is about 50 % of the variability, which is thus about 0.01 % ($0.02~\mbox{\%}\times50~\mbox{\%}$). So the energy model solar cycle estimate has the potential for being six times more accurate than the SIM solar cycle observation based only on comparison of the uncertainties. These uncertainties can be compared to the intrinsic solar cycle variability at 500 nm that is about 0.02 % for the energy result.

If the Vis–NIR energy variability result is appropriate for scaling to represent solar cycle variability, as is the case in the UV ranges, then the Vis–NIR energy variability result might be the more accurate estimate for solar cycle variability than any current or previous solar cycle measurement. This potential conclusion is the primary result from this section.

A primary concern with this potential conclusion is that the sunspot and faculae contributions may change over the solar cycle. In particular, Shapiro *et al.* (2014) show that sunspot area is much more prevalent than faculae area during higher solar activity. The energy model analysis was done over all solar activity levels, but the average of these results is weighted more toward moderate solar activity due to the nature that solar cycle maximum and minimum periods are typically about 2 years each during the ${\sim}\,11$-year solar cycle. Therefore, scaling of these energy variability results representative of moderate solar activity levels may not be appropriate for comparing to variability between true cycle maxima and minima. It is feasible that the deficit contribution as shown in Figure 9b could be higher and the excess contribution could be lower at times of high solar activity. This effect is effectively illustrated in Figure 9c, and if so, more wavelengths in the Vis–NIR ranges would have out-of-phase behavior. These concerns are only for the Vis–NIR ranges where dark sunspots (deficit) and bright faculae (excess) contributions compete for solar variability. Whereas, the variability in the UV ranges shorter than 400 nm is dominated only by the excess contribution, and the energy model variability results compare favorably with scaling of solar cycle variability observations.

## Additional Comparisons to Solar Cycle Variability

**Figure 11:**
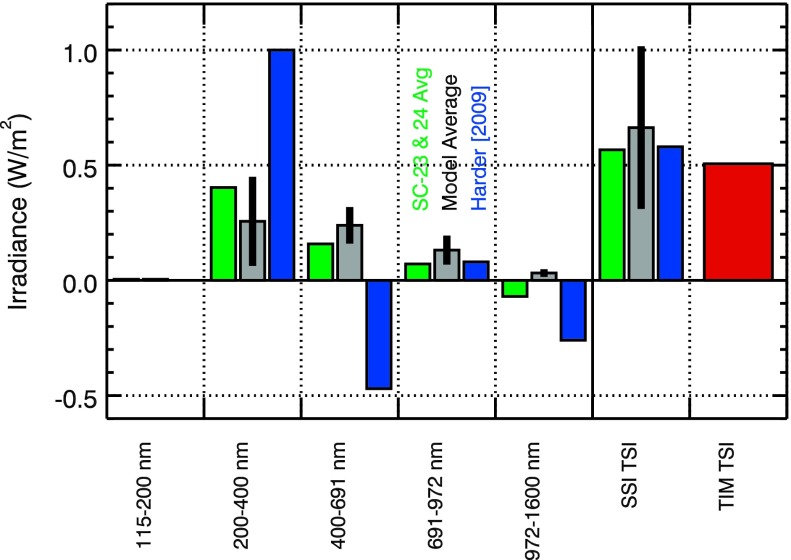
The SOLSTICE-SIM energy variability results (gray bars) and average of the SC-23 and SC-24 variability measurements (green bars) are compared to the Harder *et al.* (2009) solar cycle variability results (blue bars). The composite for the energy variability used in this figure include SORCE SOLSTICE for 115 – 270 nm and SORCE SIM for 270 – 1600 nm. The gray bars use a scaling factor of 2.0 as expected from comparing the energy variability and solar cycle variability in the FUV. The SSI TSI is the sum of all the bands’ irradiance variations. The TIM TSI is the measured TSI change between April 2004 and February 2008 (Harder’s dates), and the SSI TSI is expected to agree with the measured TSI change.

These energy variation results are compared to the Harder *et al.* (2009) broad band solar cycle results as shown in Figure 11. For this comparison, the energy relative variability (unitless) is converted to irradiance units ($\mbox{W}/\mbox{nm}/\mbox{m}^{2}$) by multiplying the energy variability by the solar cycle minimum irradiance, then this converted energy variability is integrated over wavelength into the Harder *et al.* (2009) bins. A composite of the energy variability results from multiple instruments is also made for this comparison. Our composite with the energy variability results include TIMED SEE in the 0 – 115 nm range, SORCE SOLSTICE in the 115 – 270 nm range, and SORCE SIM in the 270 – 1600 nm range. The Harder *et al.* (2009) results are the blue bars, and the scaled energy variability results are the gray bars in Figure 11.

The energy variability results need to be scaled up to the appropriate solar cycle level for this comparison. The scaling factor to match the SORCE SC-23 variability for the dates used by Harder *et al.* (2009) to the energy variability in the 130 – 150 nm range is a factor of 2.0. With this scaling factor, the energy variability composite overestimates the measured TIM TSI variation by 30 %. If the out-of-phase NIR variability of 0.02 % extends further into the IR, then the SSI TSI in Figure 11 would be reduced by $0.03~\mbox{W}/\mbox{m}^{2}$ and thus be slightly closer to the measured TIM TSI value of $0.51~\mbox{W}/\mbox{m}^{2}$. The energy variability accuracy is estimated to be about 20 % in the UV at shorter wavelengths than 250 nm, but the UV contribution to the TSI variability is very small. The estimated uncertainty for the NUV–Vis–NIR energy variability result is about 50 %. So the integrated energy spectral variability and TIM TSI variability are in agreement to within the energy model 1-$\sigma$ uncertainty.

The most notable differences between the scaled energy variability and Harder *et al.* (2009) solar cycle variability are that the MUV–NUV energy variability is three times less and that the energy variability does not have any large out-of-phase (negative) contributions in the Vis–NIR bands. Note, however, that there are out-of-phase contributions for the energy variability result in the 1400 – 1600 nm range, but the integrated contribution over the 972 – 1600 nm range is very close to zero. These energy variability results are more similar to the Lean (2000) SSI model variability, but we note that the NUV energy variability is about two times more than the Lean (2000) NUV variability that was shown in Harder *et al.* (2009).

The SORCE SC-23 and SC-24 average variability is also compared to the Harder *et al.* (2009) solar cycle results in Figure 11. These SORCE average variability results are those shown in Figures 8d and 10d and includes SOLSTICE for 115 – 310 nm and SIM for 310 – 1600 nm. As compared to the energy variability result, the SORCE average solar cycle variability is $0.2~\mbox{W}/\mbox{m}^{2}$ larger in the NUV and in the Vis–NIR bins are each about $0.1~\mbox{W}/\mbox{m}^{2}$ less. Consequently, this SSI TSI is about $0.1~\mbox{W}/\mbox{m}^{2}$ less than the energy SSI TSI and is about 15 % more than the TIM TSI variability. The differences between the SORCE average solar cycle variability and energy variability is about 50 % of the energy variability and is thus consistent within the estimated uncertainties.

The conclusions from this comparison are the following.

1. Both the energy variability and the SORCE solar cycle average variability results agree within their uncertainties with the Harder *et al.* (2009) result for the 691 – 972 nm band and SSI TSI and also with the measured TIM TSI variability.
2. The energy variability and SORCE solar cycle average variability results in the 200 – 400 nm band have less variability than the Harder *et al.* (2009) result by a factor of 3 and 2, respectively.
3. The energy variability and SORCE solar cycle average variability results in the 400 – 691 nm band are in-phase with the solar cycle *versus* being out-of-phase for the Harder *et al.* (2009) result.
4. The energy variability and SORCE solar cycle average variability results in the 972 – 1600 nm band are small contributions to the TSI variability *versus* the larger out-of-phase contribution for Harder *et al.* (2009) result.

**Figure 12:**
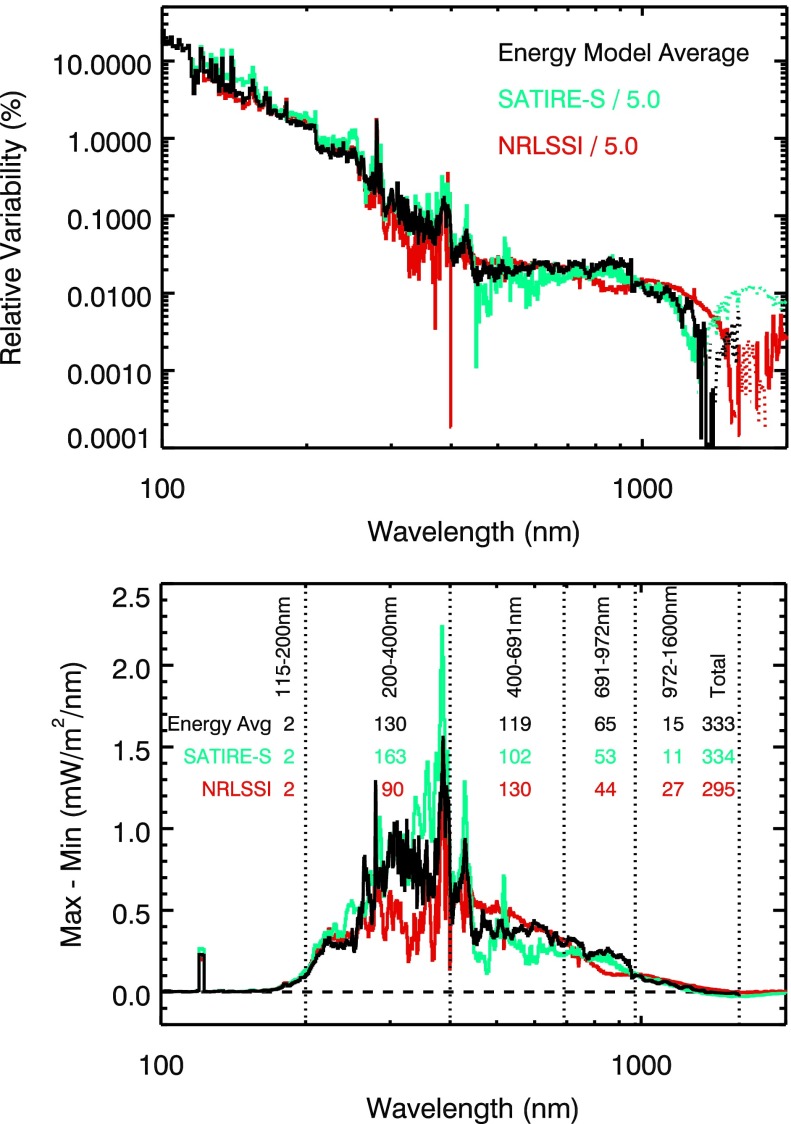
The energy variability results (black) are compared to the SATIRE-S (blue) and NRLSSI (red) model estimates for solar variability between 3-month averages at February 2002 and December 2008. The top panel shows the relative variability in percent, and the bottom panel shows the absolute variability in irradiance units. The irradiance variability (Max–Min) in broad bands is provided, and those number are in units of $\mbox{mW}/\mbox{m}^{2}$. The total variability is for the 115 nm to 1600 nm range.

Another comparison of the energy variability result is to the SATIRE-S and NRLSSI model estimates of solar cycle variability. This comparison parallels the comparison of Ball *et al.* (2014) by using 3-month averages at February 2002 and December 2008, and our comparison in Figure 12 is plotted in the same style as Figure 1 in Ball *et al.* (2014). The SATIRE-S and NRLSSI model variability estimates are scaled down by a factor of 5.0 to match the energy variability result at 150 nm. As shown in Figure 12, the energy variability results are generally in between the two model values. The NIR differences are more obvious in the relative variability plot (top panel of Figure 12), and the NUV–Vis differences are more obvious in the absolute variability (Max–Min) plot (bottom panel of Figure 12). With the energy variability results having an uncertainty of 20 – 50 %, one cannot conclude that one model agrees any better with the energy variability result than the other model.

The irradiance variability (Max–Min) in broad bands is listed in Figure 12, and those numbers multiplied by a factor of 2.0 can be compared to those in Figure 11. The conclusions listed above for Figure 11 are still consistent with the SATIRE-S and NRLSSI model comparison.

Ermolli *et al.* (2013) provide a more detailed comparison of mostly model estimates of solar cycle variability for the NUV–Vis–NIR and show significant differences between the model variability estimates and the Harder *et al.* (2009) results. Their primary concern is that the large NUV in-phase variability and large visible out-of-phase variability in Harder *et al.* (2009) are not reproduced in the NRLSSI and SATIRE models but are reproduced in the SRPM model (Fontenla et al., 2011), which is based on a negative correlation of the continuum at some wavelengths with magnetic field strength (Topka, Tarbell, and Title 1997; Foukal *et al.*^1990^). Their comparisons are similar with the differences shown here and are consistent with our conclusions listed above for the comparison with Harder *et al.* (2009). In addition to reviewing the SSI comparisons, Ermolli *et al.* (2013) review the impact of the SSI variability on Earth’s atmosphere and climate.

## Conclusions

The SORCE daily measurements for the NUV–Vis–NIR ranges have established a relatively new and unique data set for the SSI record. The SORCE SOLSTICE and SIM instruments were state-of-the-art when launched in 2003, thus the expectations are high that SORCE SSI measurements will provide the most accurate and most precise measurements of the SSI variability. Modeling of the energy variability was explored to view the SSI variability in a different way and with anticipation that its results could shed light on the SSI variability debate. As already discussed, the SSI variability from the energy technique (integration of irradiance over 6 months) has the potential to have lower uncertainties than solar cycle measurements over several years.

The following is a summary of our conclusions from this study of energy variability from modeling 6-month periods over the SORCE, TIMED, UARS, and NOAA-11 missions.

1. The energy variability method (integration of irradiance over six months) has good potential to provide a reasonable estimate of the solar cycle variability. This is confirmed in the UV 0.1 – 250 nm range through comparisons with multiple measurements and over three different solar cycles. The estimated uncertainty for the energy variability results is about 30 % of the variability for 0.1 – 290 nm and 400 – 900 nm ranges and is larger than 50 % of the variability for 290 – 400 nm and 900 – 1600 nm ranges. The energy variability results from modeling with the SFO proxies may have smaller uncertainties than those for the measured solar cycle results at some wavelengths.
2. The comparison to the model energy variability indicates that the SC-24 variability (maximum minus minimum) is about half as much as the solar cycle variability for SC-22 and SC-23.
3. The energy variability has out-of-phase contributions in the 1400 – 1600 nm range that are consistent with the NRLEUV and SATIRE models. The 2$\sigma$ uncertainty for the model fits could extend the out-of-phase behavior for the NIR range down to 900 nm. The Harder *et al.* (2009) solar cycle results included out-of-phase solar cycle variations in the 400 – 691 nm and 972 – 1600 nm ranges; the energy model results show no evidence for out-of-phase for the 400 – 691 nm range.
4. The energy variability result for the MUV–NUV range (200 – 400 nm) is about three times less than the MUV–NUV variability reported by Harder *et al.* (2009), but is about two times more than the MUV–NUV variability from the Lean (2000) SSI model.
5. New, more accurate measurements are needed for the NUV–Vis–NIR ranges.

**Figure 13:**
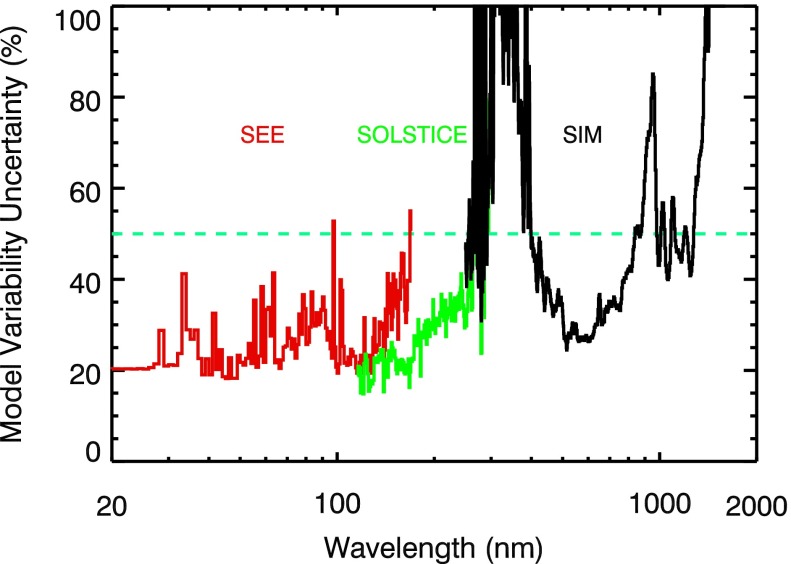
The uncertainty of the average energy variability results are shown for TIMED SEE (red), SORCE SOLSTICE (green), and SORCE SIM (black). The uncertainty has a minimum of about 20 % and larger values at wavelengths where the measurement precision is worse (SEE 170 – 195 nm, SOLSTICE 260 – 310 nm, SIM 250 – 400 nm, SIM 900 – 1000 nm) and where solar variability is small (NIR 1200 – 1600 nm).

The uncertainties of the model energy variability results range from 20 % to more than 50 % and were shown in many of the figures. For comparison over the full wavelength range, Figure 13 summarizes the uncertainties of the results presented here. It is clear from this comparison that the better results are from TIMED SEE in the 0.1 – 115 nm range, SORCE SOLSTICE in the 115 – 270 nm range, and SORCE SIM in the 400 – 900 nm range. The 290 – 400 nm range is problematic in estimating an accurate variability, and that is not only true for SORCE but also for UARS SUSIM and NOAA SBUV measurements in having variability uncertainties larger than 50 %. There are also larger than desired uncertainties for the NIR. The NIR variability is small and thus more challenging to measure and to model accurately. Because the NIR variability contribution to the TSI variability is small, it is important to focus in the near future on the NUV variability and its much larger contribution to the TSI variability.

**Figure 14:**
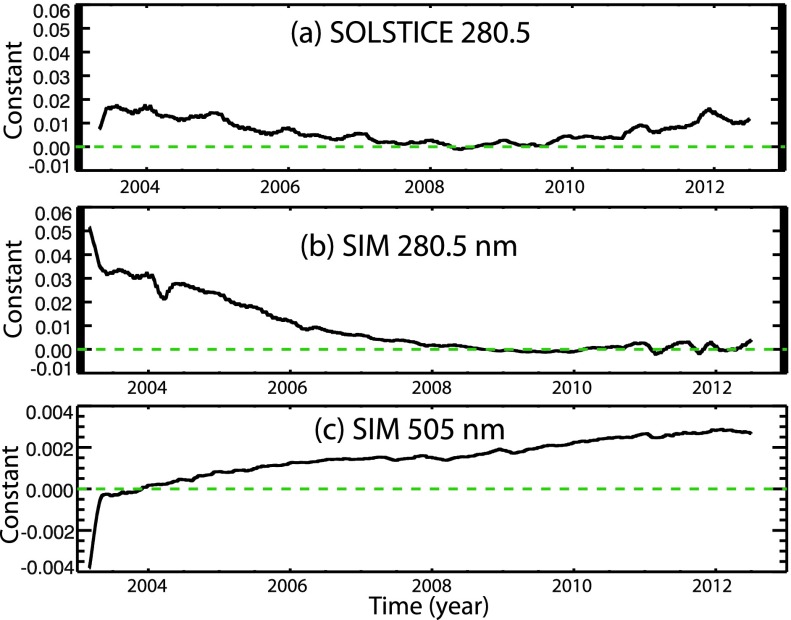
The fitted constant, the $C_{0}$ in Equation (2a), is shown for modeling the data from (a) SORCE SOLSTICE at 280.5 nm, (b) SORCE SIM at 280.5 nm, and (c) SORCE SIM at 505 nm. The constant is unitless, but it can be converted to irradiance units by multiplying it by the solar cycle minimum irradiance for its wavelength. A deviation of the fitted constant from zero can suggest a missing component of solar variability such as the case for the SOLSTICE constant trend looking like the solar cycle. A deviation from zero could also suggest an uncorrected instrument trend.

One possible approach to improve understanding of a measurement long-term record that could include a mixing of intrinsic solar cycle variations and instrument degradation trends is to examine the energy model results for the constant, $C_{0}$. As suggested in discussing the trend of the constant for the Lyman-$\upalpha$ fits in Figure 3, deviations of the fitted constant from zero might indicate instrument degradation trends and/or a need for a third component of solar variability. Examples of such trends are shown in Figure 14. The trend of the fitted constant from modeling the SOLSTICE 280.5 nm data, as shown in Figure 14a, suggests that a small solar cycle variability component is missing for the model. The SIM 280.5 nm model constant in Figure 14b may also suggest that a small solar cycle variability component is needed, but its constant trend is also suggestive of an uncorrected downward instrument trend of about 0.2 % per year. On the other hand, the SIM 505 nm model constant in Figure 14c is suggestive of a very small uncorrected upward instrument trend of about 0.03 % per year. These possible uncorrected instrument trends are within the uncertainty of the SORCE measurement accuracy, so re-analysis of the SORCE data is not anticipated to change the trending quality from that in the current SORCE data products. Nonetheless, a more detailed study of these fitted constant trends may help to guide evaluations of instrument trends and possible causes and to reveal a better understanding of solar variability and its multiple contributions.

The large differences in the NUV–Vis–NIR ranges between the energy variability technique and SORCE SSI solar cycle measurements highlights a critical need for more accurate instrumentation, despite almost four decades of SSI UV measurements. With several improvements of the TSIS SIM, such as ten times improvement for lower detector noise, ultra-clean spectrometer cavities, and three redundant channels used for degradation tracking instead of two, we anticipate that the new TSIS SSI measurements will address the lessons learned from current SSI measurements and reduce the ambiguities associated with tracking instrument degradation trends.

We plan to provide the model fitted coefficients from this study on the LISIRD web site (http://lasp.colorado.edu/home/lisird/). Additionally, we plan to release data products on LISIRD of the excess and deficit proxies that include filling the gaps in the SFO proxies, the TSI excess proxy, and extend these proxies back in time to at least 1978 using the composites of the H i Lyman-$\upalpha$ (121.6 nm) emission and TSI (Equation (4a)–(4b)).
